# Hierarchical Zeolites Prepared Using a Surfactant-Mediated Strategy: ZSM-5 vs. Y as Catalysts for Friedel–Crafts Acylation Reaction

**DOI:** 10.3390/molecules29020517

**Published:** 2024-01-20

**Authors:** Angela Martins, Beatriz Amaro, M. Soledade C. S. Santos, Nelson Nunes, Ruben Elvas-Leitão, Ana P. Carvalho

**Affiliations:** 1 Departamento de Engenharia Química, Instituto Superior de Engenharia de Lisboa, IPL, R. Conselheiro Emídio Navarro, 1, 1959-007 Lisboa, Portugal; bpamaro@gmail.com (B.A.); ruben.leitao@isel.pt (R.E.-L.); 2 Centro de Química Estrutural, Faculdade de Ciências, Institute of Molecular Sciences, Universidade de Lisboa, Campo Grande, 1749-016 Lisboa, Portugal; mssantos@ciencias.ulisboa.pt; 3 Departamento de Química e Bioquímica, Faculdade de Ciências Universidade de Lisboa, Ed.C8, Campo Grande, 1749-016 Lisboa, Portugal

**Keywords:** hierarchical Y and ZSM5 zeolites, surfactant-mediated strategy, Friedel–Crafts acylation, furan, kinetic parameters

## Abstract

Hierarchical ZSM5 and Y zeolites were prepared through a surfactant-mediated strategy with NH_4_OH changing the duration of the treatment and the amount of CTAB surfactant and taking as reference multiples of the critical micellar concentration (CMC). The materials were characterized using powder X-ray diffraction, N_2_ adsorption isotherms at −196 °C, and SEM and TEM microscopy. The catalytic performance was evaluated in Friedel–Crafts acylation of furan with acetic anhydride at 80 °C. The alkaline surfactant-mediated treatment had different effects on the two zeolites. For ZSM5, the CTAB molecular aggregates can hardly diffuse inside the medium-size pores, leading mainly to intercrystalline mesoporosity and increased external surface area, with no positive catalytic impact. On the other hand, for large-pore Y zeolite, the CTAB molecular aggregates can easily diffuse and promote the rearrangement of crystal units around micelles, causing the enlargement of the pores, i.e., intracrystalline porosity. The optimized Y-based sample, treated for 12 h with a CTAB amount 32 times the CMC, shows an increase in product yield and rate constant that was not observed when a higher amount of surfactant was added. The reuse of spent catalysts upon thermal treatment at 400 °C shows a regeneration efficiency around 90%, showing good potentialities for the modified catalysts.

## 1. Introduction

The traditional industrial processes for the synthesis of aromatic ketones, important intermediates in the production of many fine and speciality chemicals, involve abundant organic compounds, with over-stoichiometric quantities of toxic reactants as well as homogeneous catalysts operating at a high temperature. These processes originate large amounts of effluents, often leading to expensive downstream processes along with nonrecovery of valuable catalysts that are lost within the reactant effluent. Due to increasingly strict environmental legislation, nowadays, there is considerable pressure to replace these older technologies and focus on heterogeneous catalysts that can operate under mild reaction conditions and are easily recovered and reused [1,2,3].

Zeolites are good candidates to replace traditional homogeneous catalysts due to their unique combination of properties, such as ordered porosity, mechanical and thermal stability, and intrinsic acidity [4]. Despite their applications being mostly in refining and petrochemical units, further uses are also being explored in fine chemistry as catalysts or catalyst supports [5]. One of the examples is the Friedel–Crafts acylation reaction as a substitute for classic AlCl_3_ or HF homogeneous catalysts. In fact, a commercial scale process already in use for the acylation of aromatic compounds has been developed by Rhodia using BEA zeolite as catalyst [6]. However, the strict micropore nature of zeolite materials, which is an advantage in reactions where small molecules are involved, becomes a drawback when the aim is to transform large molecules because the access to the active sites may suffer severe constraints or even be hindered. To overcome this issue, several strategies have been proposed to produce zeolites that, along with micropores, also possess an additional pore system in the mesopore range, i.e., pores with widths between 2 and 50 nm [7,8,9]. These strategies can be classified as (i) bottom–up, when the synthesis protocol is modified to create mesopores, comprising hard or soft templating or even microwave synthesis, or (ii) top–down, involving the modification of a previously synthesized structure; including dealumination, desilication, and, more recently, the surfactant-templated method. 

The surfactant-templated method was originally proposed by García-Martínez and co-workers [10,11]; it comprises a post-synthesis alkaline treatment in the presence of a surfactant followed by thermal heating under autogenous pressure. When compared to desilication or dealumination, where the formed mesopores are mostly random in size and shape, this method brings the advantage of promoting the development of ordered mesoporosity. Recently, Mendoza-Castro et al. [12] critically reviewed the surfactant-templated method from the pioneer Y zeolite produced in the presence of NH_4_OH and cetyltrimethylammonium bromide (CTAB) [10] for the optimization of the operation conditions, as well as the extension of the same procedure to other zeolite structures [13,14]. Some studies also report the effect of distinctive experimental parameters, such as the effect of alternative bases like NaOH and TPAOH [13,15], or the comparative effect of surfactants with different chain lengths [16,17]. Catalytic applications of these hierarchical materials are ongoing, from the industrial hydrocracking catalysts containing surfactant-templated Y zeolite since 2013 [18] to more exploratory catalytic application, with Friedel–Crafts reactions being among them. For example, the alkylation of indole by alcohols for the synthesis of pharmaceutical compounds was studied using surfactant-templated USY, and improvements in both activity and reusability were observed when compared with commercial USY, Al_MCM-41, and Amberlyst resin [19]. More recently, the Friedel–Crafts acylation of furan by acetic anhydride using HY zeolite modified in alkaline medium in the presence of CTAB or DTAB surfactants was studied by us [16] The duration of the treatments under autogenous pressure was investigated, and it was found that in the case of CTAB, the catalytic behaviour was optimized for the sample treated during 12 h, whereas 24 h were needed for the DTAB-treated sample. Following the reasoning of the previous study and pursuing the optimized catalyst for the Friedel–Crafts acylation reaction, the purpose of this work is to study the effect of the amount of CTAB added during the autogenous treatment for a previously optimized treatment time of 12 h using multiples of the critical micellar concentration (CMC). A less-explored zeolite structure in Friedel–Crafts acylation reactions, ZSM5, was also submitted to alkaline treatment assisted by CTAB. In this case, a first set of materials was prepared to study the effect of treatment time, and, upon reaching the optimal duration, a second set was prepared by changing the surfactant amount, taking the CMC as reference. In all cases, the catalytic studies were performed using furane as the substrate and acetic anhydride as the acylating agent at 80 °C. Regeneration and reuse studies were performed for selected catalysts. 

## 2. Results

### 2.1. Materials Characterization 

ZSM5 (SiO_2_/Al_2_O_3_ = 30) and Y (SiO_2_/Al_2_O_3_ = 5.2) zeolites were submitted to basic and or acid treatments to obtain samples ZSM5_P and Y_P, which were further modified using an alkaline treatment assisted by a surfactant. This involved using commercial cetyltrimethylammonium bromide (CTAB) as surfactant in the presence of NH_4_OH solution under autogenous pressure at 150 °C. The duration of the treatment, as well as the amount of CTAB added was changed by taking as reference the critical micellar concentration (CMC). The CMC was previously determined through tensiometric and conductimetric techniques. The results obtained, 0.95 mM and 0.97 mM, respectively, are in agreement with the literature [20] 

For Y zeolite, a set of samples was prepared by changing the amount of CTAB added, taking as reference a 12 h of treatment period, which was optimized in our previous study [16]. In the case of ZSM5, two sets of samples were prepared. In the first one, the procedure described by Talebian et al. [13] was followed, and the duration of the treatment was changed. The second set took the optimized time of the first series and changed the amount of CTAB. The samples were designated as ZEO_*t*-*x*, where ZEO refers to ZSM5 or Y zeolites, *t* is the duration of the treatment (h), and *x* is the amount of CTAB, expressed as CMC multiples.

**Figure 1:**
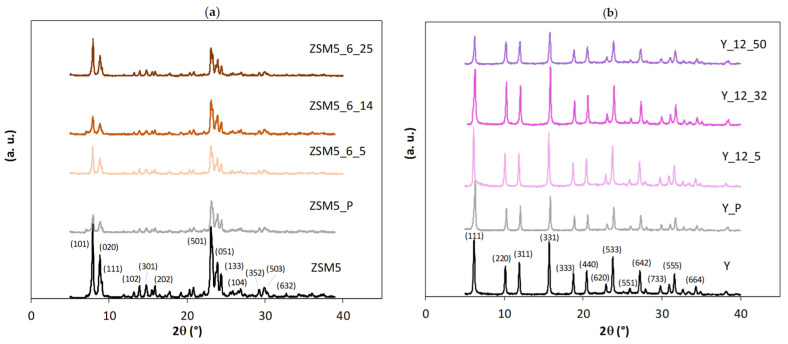
X-ray diffraction patterns of parent and modified samples through surfactant-mediated alkaline treatment for 6 h using multiples of CTAB critical micellar concentration: (**a**) ZSM5 series; (**b**) Y series. Miller indexes are attributed according to the IZA Database of Zeolite Structures (http://www.iza-structure.org/databases/ (accessed on 18 December 2023)).

The X-ray powder diffraction patterns of parent and selected modified samples (CTAB amount series) are presented in Figure 1. In all cases, the diffractograms show a long-range crystal ordering, meaning that the crystal structure was kept. To quantify some loss of crystallinity as consequence of the treatments, the degree of crystallinity (C*_XRD_*) was calculated following the procedures reported in ASTM D 5758-01 [21] and ASTM 3906-03 [22] in the case of, respectively, ZSM5- and Y-derived samples. In both cases, the starting material was taken as a reference, and the relative intensity of the peaks between 22.44 and 25.15 °2θ (ZSM5-related samples) and the peaks corresponding to the Miller indexes (331), (333), (440), (533), (642), and (664) (Y-related samples) was assessed by considering the peak integration obtained using “Peak-fit” 11.0 version software. The percentage of crystallinity, *C*_XRD_, for all samples is displayed in Table 1 and Table 2.

When comparing results for the modified samples prepared from ZSM5 and Y zeolites, a more significant loss of crystallinity is verified for the ZSM5-based materials, where values lower than 50% were generally obtained for both series. This behaviour can be attributed to the severe pre-treatment conditions used to produce the ZSM5_P sample, which is in line with what was already observed by Talebian et al. [13]. For Y-based materials, the pre-treated sample Y_P and surfactant-modified materials present crystallinity values mostly in the range of 56–67%, except for Y_12_50, i.e., the sample prepared using the higher amount of CTAB, which retained only 47% of the structural order of the parent material.

**Figure 2:**
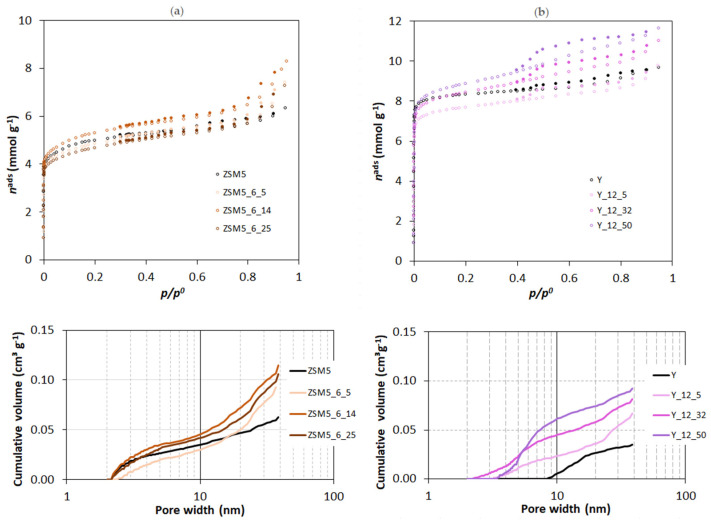
N_2_ adsorption isotherms at −196 °C (**top**) and mesopore size distribution (**bottom**) of parent and modified samples through surfactant-mediated alkaline treatment for 6 h using multiples of CTAB critical micellar concentration: (**a**) ZSM5 series; (**b**) Y series. In the isotherms, open and closed symbols represent, respectively, adsorption and desorption experimental points.

**Figure 3:**
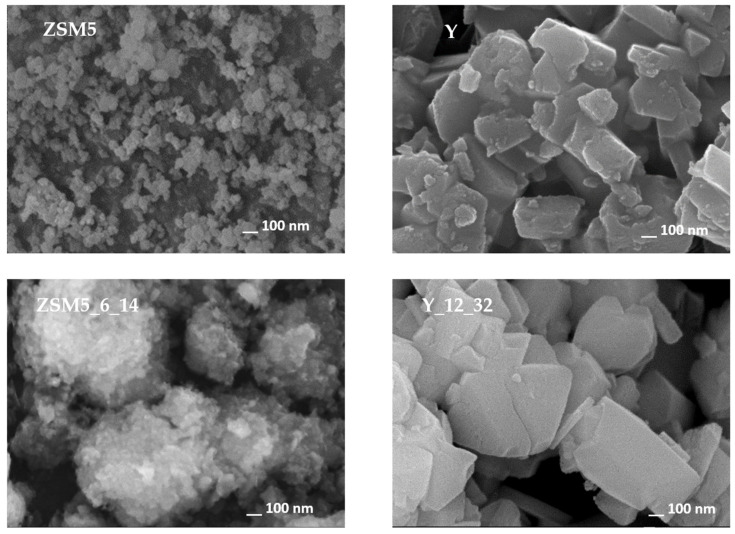
SEM images of parent and selected treated samples.

The N_2_ adsorption isotherms at −196 °C reproduced in Figure 2 are representative of the results obtained in the present study. In all cases, the curves can be classified as type I+IV isotherms [23], thus revealing the intrinsic micropore nature of the samples allied with a mesopore network. In the case of the starting ZSM5 and Y structures, the presence of the mesoporosity is interpreted as the result of the aggregation of the small crystals (see SEM images reproduced in Figure 3). The curves obtained with the ZSM5-modified materials do not reveal any important modification in the low relative pressure region, so no important change in the microporosity occurred. On the contrary, a pronounced upward deviation at the higher relative pressure region is observed, indicating the presence of an important mesopore network, which was expected as a consequence of the treatments applied. The curves for the Y-modified materials show a different pattern because the upward deviation starts at quite low relative pressure (around 0.2–0.3), suggesting that, in this case, the most important textural modification must be the development of wider micropores and/or narrow mesopores. Regarding the hysteresis loop, all the curves present an H4 loop.

To quantify the textural parameters, the isotherms were analyzed by applying the α_s_ method, taking as reference the isotherm obtained in a non-porous silica reported in ref [24]. A typical α_s_ plot presents two linear regions: one that allows us to quantity the volume of the narrow micropores, which are characteristic of the zeolite structure, i.e., ultramicropores (φ < 0.7 nm), and a second one that allows for the estimation of the total micropore volume, which includes the volume of the ultra- and supermicropores (0.7 nm < φ < 2 nm). The ultramicropore volume, *V*_ultra_, is obtained through the back extrapolation of the linear region defined by the experimental points determined between *p*/*p*^0^ > 0.02 and, normally, *p*/*p*^0^ = 0.4 (i.e., α_s_ = 1). The back extrapolation of the region defined by the points obtained at *p*/*p*^0^ > 0.4 corresponds to the total micropore volume, *V*_micro_, thus allowing for the estimation of the supermicropore volume, *V*_super_, through the difference *V*_micro_ − *V*_ultra_. From the slope of the latter linear region, it is also possible to estimate the value of the external area, *A*_ext_. The mesoporous volume (*V*_meso_) is obtained through the difference between *V*_total_ and *V*_micro_, where *V*_total_ is the total pore volume, corresponding the total amount of N_2_ uptake at *p*/*p*^0^ = 0.95, according to the Gurvich rule [24].

The textural parameters obtained from the analysis of the isotherms according to the methodology described in the last paragraph are quoted in Table 1 and Table 2 for the samples modified from ZSM5 and Y, respectively. The analysis of the values obtained makes clearer the different textural evolution of the two structures submitted to CTAB-assisted alkaline treatment. In fact, in the case of ZSM5 samples, the most expressive change is related to the increase in the mesopore volume, which, in some cases, is more than twice the value presented by the starting sample, as in the case of ZSM5_P, clearly showing that the textural modifications already occur during the pre-treatment steps. The subsequent CTAB-assisted treatment in the presence of NH_4_OH promotes, in some cases, an increase in *A*_ext_. In the Y-derived materials, the mesopore volume remains almost unchanged, but there is an increase in the values of *V*_super_, especially in the case of samples treated with higher amounts of CTAB. It must also be noted that besides the characteristics previously discussed, the N_2_ isotherms of the Y-modified samples have a somewhat pronounced “step” at around 0.4 > *p*/*p*^0^ > 0.6. This result has been reported in the literature, and, in a previous study [16], we interpreted the changes that this configuration implies in the α_s_ plots by considering that the back extrapolation of the linear region defined by the experimental values obtained at higher relative pressures, in this case quantifies the micropore volume and also the volume of narrow mesopores because it starts with points corresponding to α_s_ = 1.2 (i.e., *p*/*p*^0^ ≈ 0.6). So, for Y-modified samples, the value of *V*_super_ corresponds to the volume of supermicropores + narrow mesopore, which increases upon treatment, showing that this is the most important textural change, as suggested by the analysis of the isotherms’ configuration. 

The mesopore size distribution obtained using Hybrid Density Functional Theory (DFT Plus^®^ V2.01 ASAP 2010 V5.00) considering pores with a cylindrical shape is also displayed in Figure 2 (bottom), and it corroborates the previous interpretation. In these plots, it is quite clear that in relation to the parent structures, ZSM5-modified samples have a larger volume of mesopores wider than 20 nm (all the cumulative N_2_ uptake volume curves present an accentuated upward deviation). On the other hand, the plot corresponding to the Y series is completely different because while the starting sample presents few mesopores and all are above 10 nm wide, all the modified samples have the most important increase in the cumulative volume of N_2_ uptake in pores up to that value, particularly sample Y_12_32, which presents narrower mesopores (that is, pores below ~3.5 nm). After 10 nm, the curves are almost parallel to that of the starting sample, indicating that the wider mesopores are those already present in the starting sample. 

CTAB micellar aggregates have been used as soft templates in materials science targeting induced mesoporosity in zeolites [7]. The G-Martínez group has extensively studied surfactant-induced mesoporosity in zeolites and advocate that this approach is limited to small-headed, single-chain surfactants that, driven by electrostatic interaction with the Si-O^−^ generated in the base pretreatment step, would diffuse through zeolite crystals, thus aggregating within the brittle zeolite structure [17,25]. Tensiometry is frequently used to estimate surfactants’ head group area on a packed air/water interface, and values between 0.55 nm^2^ and 0.76 nm^2^ per molecule are found in the literature [26], with values affording a head group diameter between 0.20 and 0.24 nm, i.e., capable of diffusing within even small-pore-size zeolite structures. However, under the experimental conditions used, which is a CTAB concentration in the range of 5 to 50 times the CMC in 3 M NH_4_OH, most surfactant will be aggregated, so the molecular entities present are much larger. Indeed, small-angle neutron scattering (SANS) revealed that CTAB forms ellipsoidal aggregates with a 2.4 to 2.6 nm minor axis and a variable major axis, which increases with increasing CTAB concentration and spans from 4.7 to 8.2 nm [26,27]. The mesopore size distribution for the samples upon pre-treatment, ZSM5_P and Y_P (see Figure S1), evidences that for zeolite ZSM5, a significant fraction of the mesopore volume pertains to pores smaller than 10 nm, thus hindering or eventually preventing the diffusion of CTAB molecular aggregates. For zeolite Y, the mesopore volume profile is completely different, with most pores being larger than 10 nm and therefore enabling the diffusion of surfactant molecular aggregates through the brittle zeolite structure, thus affording a zeolite structure reconstruction around the ellipsoidal worm-like aggregates. 

The morphology of parent and selected modified samples was evaluated through scanning electronic microscopy (SEM). The images displayed in Figure 3 clearly show the distinct crystal sizes and shapes of parent ZSM5 and Y zeolites, with aggregates composed of small crystals for Y zeolite and even smaller crystals in the case of ZSM5 material.

**Figure 4:**
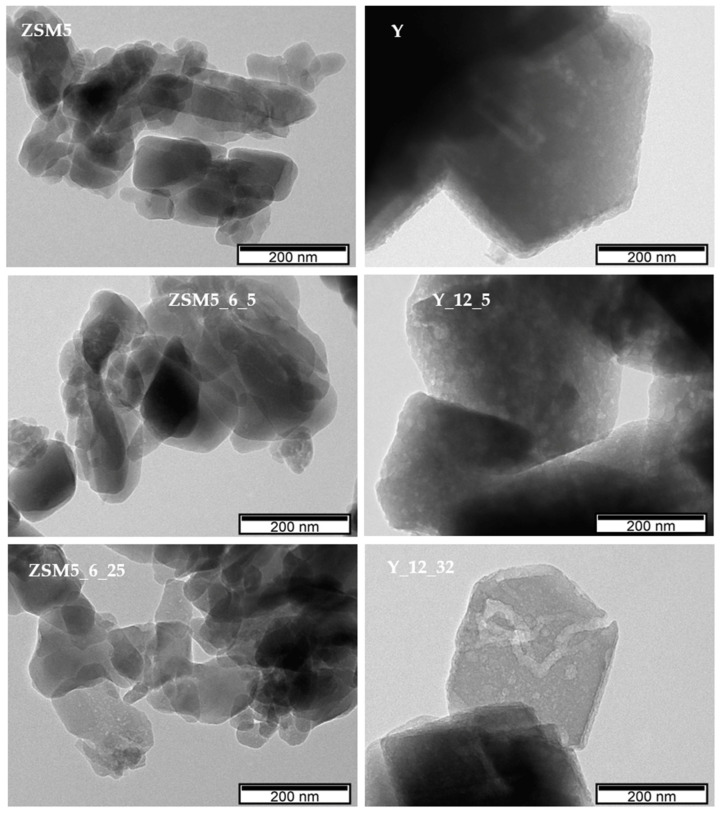
TEM images of parent and selected treated samples.

Regarding the images of selected modified samples, in the case of ZSM5_6_14, large aggregates can be visualized, which are composed of smaller crystals when compared with the parent material. For the Y_12_32 sample, the crystals are also presented as aggregates while keeping the morphology and size of the parent material, indicating that the modifications that occurred because of the alkaline treatment assisted by CTAB occurred mainly inside the zeolite crystals, in line with the previous analysis of textural parameters. A deeper visualization of the crystal modifications was complemented using transmission electronic microscopy (TEM), and the images are presented in Figure 4 for selected samples. As expected, the ZSM5 image shows small overlapping crystals. Upon NH_4_OH + CTAB treatment, both ZSM5_6_5 and ZSM5_6_25 show some lighter zones as well as some fragmentation of the crystals, especially at the edges, which can be attributed to the severity of the pre-treatment, allowing the occurrence of desilication and/or dealumination of the zeolite structure, in agreement with the previously discussed decrease in crystallinity and increase in *A*_ext_ (see Table 1). However, the alkaline + CTAB treatment seems to be relevant because in the case of the ZSM5_6_25 sample, where the amount of surfactant added during the treatment is higher, the presence of lighter zones is more visible. For parent Y and selected modified samples, a completely different scenario is presented because the crystals remain intact upon treatments. However, lighter zones are regularly distributed along the inside of the crystals in sample Y_125, suggesting that the enlargement of the porosity occurred because of the alkaline treatment assisted by CTAB. However, as the amount of CTAB added increases from 5 to 32 times the CMC, some peculiar tunnel-shaped pores appear in the Y_12_32 sample, mirroring the increase in the longer ellipsoidal radius into a worm-type structure [28,29] or even an (NH_4_OH)-induced rod transition at lower CTAB concentrations.

### 2.2. Catalytic Tests and Kinetic Modeling

**Figure 5:**
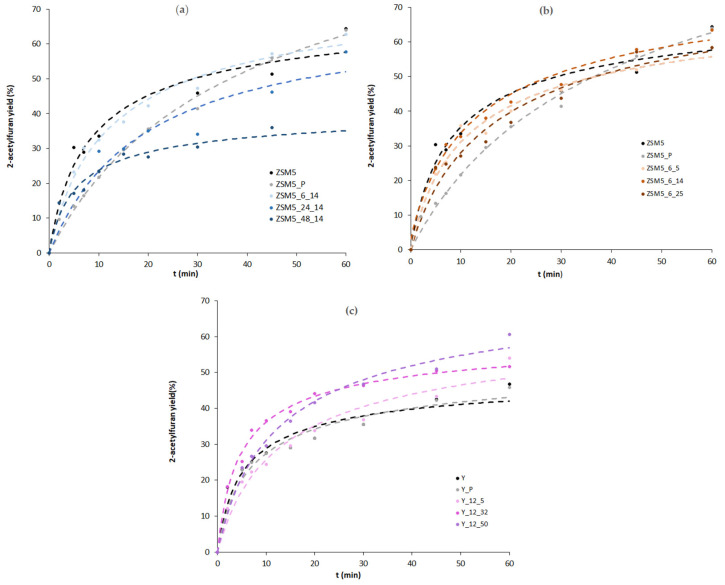
Product yield as a function of reaction time for the effect of treatment duration on ZSM5 (**a**) for the effect of the amount of CTAB on ZSM5 (**b**) and for the effect of the amount of CTAB on Y (**c**). (The dashed curves represent calculated values resulting from the application of the kinetic model).

The catalytic behaviour of parent and modified materials was studied in a Friedel–Crafts acylation reaction using furan as substrate and acetic anhydride as acylating agent at 80 °C. Figure 5 shows, for selected samples, the product yield, considering that the reaction is almost complete into 2-acetylfuran due to the high stability of the α-intermediate. To complement the analysis of yield vs. reaction time curves, the kinetic parameters obtained, from the non-linear regression treatment, applied to the simplified Langmuir–Hinshelwood kinetic model (see Equation (2) in Section 3) were calculated, and are displayed in Table 3 and Table 4. The relevant statistical parameters present good statistical criteria, i.e., high values of R^2^ and F and low S_Fit_. A quick inspection of the curves displayed in Figure 5 shows a sharp increase in the first 10 min of the reaction for parent and modified samples, followed by a slope attenuation for longer reaction times; however, the catalytic behaviour is distinct for the ZSM5- and Y-modified materials. 

For the ZSM5-based materials, both time and CTAB amount series present no gain in catalytic performance. Indeed, a significant decrease in 2-acetylfuran yield is obtained for the pre-treated sample, ZSM5_P, as well as a lower rate constant, *k*, and relative sorption equilibrium constant, *K*_r_, because of the severe alkaline + acid pre-treatment that led to an important loss of crystallinity as well as a, most likely, reduction in the acid sites concentration due to the elimination of the Si or Al atoms from the zeolite framework, which, according to Talebian et al. [13], can achieve over 30%. Concerning the effect of the subsequent alkaline + surfactant treatment, as can be observed from Figure 5a,b, the yield vs. reaction time curves are always below the one for the parent ZSM5, which is in line with the rate constant values in Table 3. Although the textural data in Table 1 show some development of mesoporosity, especially for samples ZSM5_3_14 and ZSM5_12_14, the alkaline treatment assisted by CTAB did not improve the catalytic performance of the modified samples, probably because the controlled intracrystalline enlargement of the pores, typically attributed to the crystal rearrangement promoted by the surfactant micelles, did not occur because the pore sizes of ZSM5 hinder the diffusion of CTAB aggregates inside the zeolite crystals. Instead, large crystal agglomerates tend to form (see SEM images in Figure 3), probably around the surfactant micellar aggregates, which is also attested by the increase in the *A*_ext_ values. 

Concerning the Y-based samples, a different scenario is presented. The milder pre-treatment performed on the Y_P sample almost gives an overlap in the yield vs. reaction time curves as well as identical *k* and *K*_r_, indicating that the crystals kept their integrity, although some loss of crystallinity occurred. For the alkaline + surfactant-modified samples, the product yield vs. time curves and kinetic parameters do not present any regular trend with the increase in the amount of surfactant added during the alkaline treatment. Indeed, only sample Y_12_32 clearly presents higher product yield during all of the reaction time, which can be translated into a higher value of *k*. The singular performance of this sample may be ascribed to the increase in *V*_super_ and *V*_meso_. In fact, the larger micropores/small mesopores that seem to have a crucial effect on the catalytic performance can be visualized in the mesopore size distribution (Figure 2), denoting an improved molecular diffusion inside the internal porosity of the zeolite samples. This observation attests the effect of the enlargement of the micropores/small mesopores on upgrading the catalytic performance, whereas large mesopores or intercrystalline mesoporosity do not seem to give higher product yields in this type of reaction, which is in line with what was already found in our previous study [16].

### 2.3. Regeneration Assays

**Figure 6:**
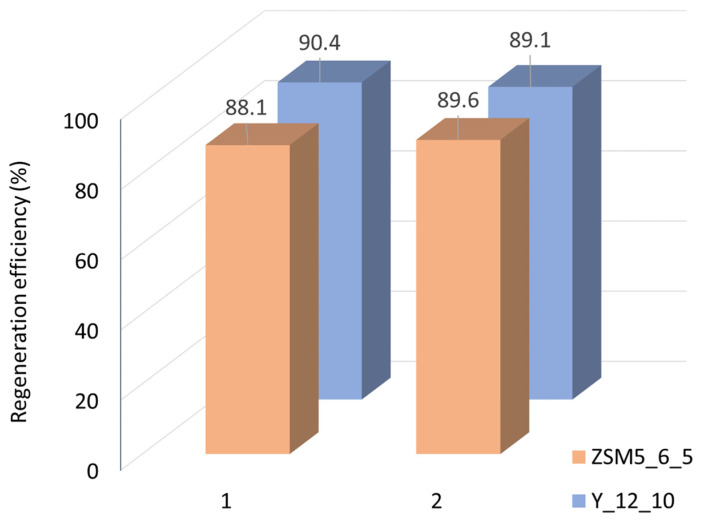
Regeneration efficiency for two consecutive recycling assays.

The ability to reuse the spent catalysts was investigated by selecting two samples of each material that stood out for their good catalytic performance: ZSM5_6_5 and Y_12_10. After a first catalytic run, the spent catalysts were submitted to two consecutive catalytic runs under the same reaction conditions upon regeneration at 400 °C for 4 h, in a muffle, after each run. For both catalysts, the regeneration efficiency (Figure 6) is around 90%, thus keeping the same efficiency between the two cycles.

Thermogravimetric (TG) analysis was performed for the ZSM5_6_5 sample after the catalytic experiments on the fresh sample and upon the two regeneration cycles to investigate if the thermal treatment conditions were enough to efficiently remove the species adsorbed during the catalytic assays. The weight loss associated with the decomposition of the species retained inside the pores as a function of temperature for fresh and spent catalyst presents in all cases a major weight loss between 7 and 8% until 400 °C and less than 1.5% between 400 and 550 °C. These results are in line with the regeneration efficiency (Figure 6), confirming that the thermal treatment conditions are adequate to allow the reuse of the catalysts.

## 3. Materials and Methods

### 3.1. Catalysts Preparation

ZSM-5 zeolite (MFI structure) with SiO_2_/Al_2_O_3_ = 30 and Y zeolite with SiO_2_/Al_2_O_3_ = 5.2 were both supplied by Zeolyst (CBV 3024E Lot. 2200-99 and CBV500 Lot. 50006N003228, respectively). The materials were purchased in ammonium form and converted into the protonic form through calcination under dry air in a muffle (Nabertherm B170, Bahnhofstr, Germany) at 550 °C (heating rate 5 °C min^−1^) for 4 h, therein designated as ZSM5 and Y for simplicity. The chemicals used for zeolite treatments and for the catalytic experiments were acquired from Merck (Darmstadst, Germany) and were used as received without further purification. The zeolite samples were modified through surfactant-mediated alkaline treatment. Two sets of ZSM5-derived samples were prepared to study (i) the effect of treatment duration and (ii) the influence of the amount of CTAB added. Prior to the alkaline surfactant-assisted treatment, ZSM5 was submitted to a pre-treatment to sensitize the zeolite structure, following the procedure described in ref. [13]. In brief, ZSM5 was suspended in 0.25 M NaOH at 80 °C for 1 h, followed by an acid leching with 0.6 M H_2_SO_4_ solution at 80 °C for 3 h, using a ratio of volume solution/weight zeolite of 4, giving the ZSM5_P sample. To study the effect of treatment duration, the ZSM5_P sample was suspended in 0.3 M NH_4_OH, using the ratio of 64 mL of solution per 1 g of zeolite, and a fixed amount of CTAB (0.3 g) was added, corresponding to 14 times the CMC. The suspension was stirred for 20 min at 25 °C, transferred to a PTFE coated stainless steel autoclave, and heated at 150 °C under autogenous pressure for 6 to 48 h. To evaluate the influence of the CTAB amount, the same procedure was followed, but the amount of CTAB added was changed using multiples of CMC: 5, 10, 14, or 25. In all cases, the solids were recovered through centrifugation, dried overnight at 80 °C, and calcined at 550 °C for 3 h (heating ramp of 2 °C min^−1^). The materials were designated as ZSM5_*t*_*x*, where *t* is the duration of the treatment and *x* represents the concentration of CTAB expressed as multiples of CMC. For Y-based samples, the parent material was pre-treated with a 10 wt.% citric acid solution using the ratio of 1 g of zeolite per 1 mmol of citric acid [10,15,30], giving the Y_P sample. Upon washing and drying, this sample was suspended in a 0.37 M NH_4_OH using the ratio of 64 mL of solution per 1 g of zeolite, and then different amounts of CTAB were added using multiples of CMC: 5, 10, 25, 32, or 50. The suspensions were transferred to a PTFE coated stainless-steel autoclave and heated at 150 °C during the previously optimized time of 12 h [16]. The obtained materials were labeled Y_12_*x*, where *x* refers to the concentration of CTAB expressed as multiples of CMC. To ensure that after the alkaline CTAB-assisted treatments the samples were in full protonic form, an ion exchange procedure was performed with a 2 M NH_4_NO_3_ solution using a ratio of 25 mL for 1 g of zeolite at 80 °C for 6 h. The materials were recovered through centrifugation, dried, and calcined at 550 °C (heating rate 5 °C min^−1^) for 4 h.

### 3.2. Physicochemical Characterization

The determination of CMC for the CTAB surfactant was quantified through tensiometry (Kruss K100 Tensiometer, Hamburg, Germany) and conductivity (Radiometer, MeterLab CMD 230, Copenhagen, Denmark) measurements. The structural characterization of the zeolite materials was made through X-ray powder diffraction (XRD) patterns obtained at room temperature in a Pan’Analytical PW3050/60X’Pert PRO (θ/2θ) diffractometer (Phillips, Almelo, The Netherlands) equipped with the X’Celerator detector with automatic data acquisition (X’Pert Data Collector (v2.0b) software) and using monochromatized CuKα radiation as the incident beam, 40 kV–30 mA. Diffractograms were obtained through continuous scanning in a 2θ range of 5°–40° with a step size of 0.017 °2θ and a time per step of 0.6 s. Scanning and Transmission Electronic Microscopy (SEM and TEM) were carried out in Hitachi (Chiyoda, Japan) model S400 (SEM) and H-8100 (TEM) microscopes. The textural characterization was obtained through low-temperature N_2_ adsorption isotherms obtained in an automatic apparatus, ASAP 2010 (Micromerics Instruments Corporation, Norcross, GA, USA). Prior to the adsorption measurements, about 50 mg of zeolite samples was outgassed at 300 °C for 3 h under a vacuum greater than 10^−2^ Pa.

### 3.3. Catalytic Tests

The experiments were performed in a three-neck, round-bottom flask equipped with a reflux condenser placed in a heating plate equipped with temperature control (IKA C-MaHS7, Staufen, Germany). Typically, furan (0.71 g, i.e., 10.5 mmol) was added to acetic anhydride (5.5 g, i.e., 52.5 mmol), obeying a molar ratio between substrates and the acylating agent equal to 5. Then, 150 mg of zeolite samples was added and heated to 80 °C. Periodically, less than 0.5 mL of the samples of the reaction mixture was taken using a hypodermic syringe (Millipore Swinnex support with a Millipore Durapore 0.45 μm). Upon quenching in an ice bath, each aliquot was analyzed in a gas chromatograph (Perkin Elmer Auto System, Norwalk, CT, USA) with an FID detector, using N_2_ as a carrier gas in a 30 m DB-5MS capillary column (Agilent, Santa Clara, CA, USA) The temperature of the GC injector was 250 °C, and it was 275 °C for the detector. The GC oven for the analysis started at 45 °C for 5 min, followed by a temperature increase of 10 °C min^−1^ until 200 °C before staying at this temperature for 10 min. All products and unconverted reactants were identified through comparison of the retention times with previously injected standards, and the signals were integrated using DataApex Clarity 9.0 version Software (DataApex, Prague, Czech Republic).

### 3.4. Kinetic Studies

In line with our previous study [31], it is assumed that the Langmuir–Hinshelwood model is the one that best describes the liquid-phase reaction that occurs in the presence of porous materials. This model accounts for the competition between reactants and products for the active sites located inside the pores of the catalysts and admits that competition between the reacting species for those active sites can lead to obstruction of the inner pores, thus resulting in the deactivation of the catalyst [32,33].

*A*_(ads)_ + S_(ads)_ → *P*_(ads)_ (1)

where *A* is the acylating agent, *S* is the substrate, and *P* is the acylated product. Assuming the same methodology as reported previously [30], the reaction rate can be expressed, upon simplification, as:

(2) $$r≅\frac{k\left[A\right]\left[S\right]}{{\left(\left[A\right]+\left[S\right]+K_{r}\left[P\right]\right)}^{2}}$$

where *k* is the rate constant of the rate-determining step and *K*_r_ is the ratio between the adsorption equilibrium constant of the products(s) and the normalized equilibrium constant of the reagents. The values corresponding to *k* and *K*_r_ and the related statistical parameters are estimated using nonlinear regressions with Table Curve 2D software 5.0.1.

### 3.5. Recycling Tests

The recyclability of the catalysts ZSM5_6_5 and HY_12_10 was investigated through reuse in two consecutive cycles. A new cycle was initiated after the previous one after the addition of new typical portions of furan and acetic anhydride. The reactions’ products were analyzed as mentioned above, and the catalysts were recovered through filtration, dried overnight at 60 °C, and submitted to a thermal treatment under dry air in a muffle (Nabertherm B170, Bahnhofstr, Germany) at 400 °C for 4 h with a heating rate of 5 °C min^−1^. TGA experiments were carried out in the range of 35–550 °C on a Perkin Elmer (Norwalk, CT, USA) TGA7 apparatus. The sample purge and the balance chamber were kept under a nitrogen flow (Praxair 5.0) of 22.5 cm^3^ min^−1^ and 38 cm^3^ min^−1^, respectively. The mass scale of the instrument was calibrated with a standard 100 mg weight, and the temperature calibration was based on the measurement of the Curie points (T_C_) of alumel alloy (Perkin-Elmer, T_C_ = 154 °C) and nickel (Perkin-Elmer, mass fraction 0.9999, T_C_ = 355 °C) standard reference materials. The Pyris V. 7.0.0.0110 software package was used for instrument control and data acquisition.

## 4. Conclusions

Hierarchical ZSM5 and Y zeolites were obtained through a surfactant-mediated strategy in the presence of NH_4_OH, aiming to explore the potentialities of these materials as heterogeneous catalysts in Friedel–Crafts acylation reactions. In the case of ZSM5 samples, even the hard pre-treatment conditions did not allow for efficient diffusion of CTAB molecular aggregates inside the medium-size pores of this zeolite structure. Accordingly, large clusters composed of very small crystals were formed, probably around CTAB aggregates, producing materials with textural properties that do not differ significantly from the pre-treated sample, with no positive impact on the catalytic performance.

In the case of Y samples, the large pores, typical of the Y zeolite structure, allowed for the efficient diffusion of CTAB molecular aggregates that, for high amounts of added CTAB, evolve into a worm-type structure, especially in the case of the Y_12_32 sample, leading to an increase in product yield and rate constant. However, when more CTAB was added, the same trend was not observed despite the higher *V*_super_ + *V*_meso_, which can be explained by the significant loss of crystallinity.

For both ZSM5- and Y-modified selected samples, the reuse studies showed an efficiency of around 90% after two consecutive regeneration assays at 400 °C and good thermal stability; thus, they demonstrate the potential of the modified materials to be used as catalysts for Friedel-Crafts acylation reactions.

## Figures and Tables

**Table 1:** Degree of crystallinity, *C*_XRD_, and textural parameters obtained from N_2_ adsorption isotherms: ultra and supermicropore volumes, *V*_ultra_ and *V*_super,_ respectively, mesopore volume, *V*_meso_, and external surface area, *A*_ext_, of parent zeolite ZSM5 and modified samples through surfactant-mediated alkaline treatment (effect of treatment period and amount of CTAB).

Sample	*C*_XRD_ (%)	*V*_ultra_ (cm^3^ g^−1^)	*V*_super_ (cm^3^ g^−1^)	*V*_meso_ (cm^3^ g^−1^*)*	*A*_ext_ (m^2^ g^−1^)
ZSM5	100	0.12	0.03	0.07	42
ZSM5_P	47	0.11	0.02	0.15	72
Effect of duration of treatment					
ZSM5_3_14	49	0.12	0.01	0.16	82
ZSM5_6_14	50	0.12	0.02	0.14	73
ZM5_12_14	51	0.12	0.02	0.15	78
ZSM5_24_14	49	0.12	0.01	0.15	77
ZSM5_48_14	47	0.12	0.01	0.12	50
Effect of amount of CTAB					
ZSM5_6_5	43	0.10	0.02	0.12	55
ZSM5_6_14	50	0.12	0.01	0.14	73
ZSM5_6_25	41	0.10	0.04	0.12	62

**Table 2:** Degree of crystallinity, *C*_XRD_, and textural parameters obtained from N_2_ adsorption isotherms: ultra and supermicropore volumes, *V*_ultra_ and *V*_super_, respectively, mesopore volume, *V*_meso_, and external surface area, *A*_ext_, of parent zeolite Y and modified samples through surfactant-mediated alkaline treatment using different amounts of CTAB.

Sample	*C*_XRD_ (%)	*V*_ultra_ (cm^3^ g^−1^)	*V*_super_ ^1^ (cm^3^ g^−1^)	*V*_meso_ (cm^3^ g^−1^*)*	*A*_ext_ (m^2^ g^−1^)
Y	100	0.26	0.04	0.08	51
Y_P	61	0.22	0.04	0.08	41
Y_12_5	60	0.22	0.04	0.09	43
Y_12_10	67	0.24	0.05	0.08	40
Y_12_25	56	0.22	0.06	0.09	53
Y_12_32	67	0.22	0.07	0.10	56
Y_12_50	47	0.22	0.09	0.09	65

^1^ For all but the Y sample, the values of *V*_super_ quantify the volume of supermicropores (0.7 nm < ϕ < 2 nm) and narrow mesopore (see details in the text).

**Table 3:** Rate constants (*k*) and relative sorption equilibrium constants (*K*_r_) of Friedel–Crafts acylation reaction of furan with acetic anhydride for ZSM-5-based catalysts. Also presented are the fits’ statistical figures of merit: determination coefficient (*R*^2^) regression standard deviation of fit (*s*_fit_) and Fisher–Snedecor parameter (*F*).

Catalyst	*k* (mmol min^−1^ g^−1^)	*K*_r_	*R* ^2^	*S*_fit_	*F*
ZSM5	7.3 ± 0.4	16.1 ± 2.6	0.975	0.064	198
ZSM5_P	2.27 ± 0.06	6.3 ± 0.5	0.987	0.011	811
Effect of duration of treatment					
ZSM5_3_14	3.8 ± 0.2	13.7 ± 2.1	0.978	0.031	184
ZSM5_6_14	6.7 ± 0.5	23.0 ± 3.3	0.979	0.032	290
ZSM5_12_14	6.4 ± 0.4	47.2 ± 6.9	0.988	0.038	400
ZSM5_24_14	2.7 ± 0.2	11.9 ± 2.2	0.948	0.031	90
ZSM5_48_14	6.2 ± 0.5	35.1 ± 6.6	0.938	0.073	106
Effect of amount of CTAB					
ZSM5_6_5	5.2 ± 0.2	15.9 ± 1.8	0.976	0.036	287
ZSM5_6_14	6.4 ± 0.4	47.2 ± 6.9	0.979	0.032	290
ZSM5_6_25	3.9 ± 0.3	11.9 ± 1.8	0.950	0.038	134

**Table 4:** Rate constants (*k*) and relative sorption equilibrium constants (*K*_r_) of Friedel–Crafts acylation reaction of furan with acetic anhydride for Y-based catalyst. Also presented are the fits’ statistical figures of merit: determination coefficient (*R*^2^) regression standard deviation of fit (*S*_fit_) and Fisher–Snedecor parameter (*F*).

Catalyst	*k* (mmol min^−1^ g^−1^)	*K*_r_	*R* ^2^	*S*_fit_	*F*
Y	7.9 ± 0.6	29.1 ± 5.5	0.937	0.089	118
Y_P	8.1 ± 0.3	30.1 ± 5.0	0.988	0.042	582
Y_12_5	4.1 ± 0.3	16.6 ± 2.6	0.938	0.045	121
Y_12_10	5.9 ± 0.4	11.8 ± 1.8	0.956	0.056	154
Y_12_25	5.2 ± 0.3	15.4 ± 2.0	0.967	0.043	206
Y_12_32	10.5 ± 0.6	23.9 ± 3.2	0.972	0.079	284
Y_12_50	5.8 ± 0.2	17.5 ± 1.8	0.981	0.036	362

## Data Availability

Data are contained within the article and Supplementary Materials.

## Supplementary Materials

The following supporting information can be downloaded at: https://www.mdpi.com/article/10.3390/molecules29020517/s1, Figure S1: Mesopore size distribution of pre-treated samples ZSM5_P and Y_P.
