# Supplementary material for: Hierarchical Zeolites Prepared Using a Surfactant-Mediated Strategy: ZSM-5 vs. Y as Catalysts for Friedel–Crafts Acylation Reaction
Source: Molecules. 2024 Jan 20;29(2):517. doi: 10.3390/molecules29020517 (PMC10818646; doi:10.3390/molecules29020517)
Supplement: Supplementary file 1 [file molecules-29-00517-s001.zip › molecules-2810405-supplementary.pdf]

molecules

Hierarchical Zeolites Prepared Using a Surfactant-Mediated Strategy: ZSM-5 vs. Y as Catalysts for Friedel–Crafts Acylation Reaction

Angela Martins [1,2,*], Beatriz Amaro [1], M. Soledade C. S. Santos [2,3], Nelson Nunes [1,2], Ruben Elvas-Leitão [1,2] and Ana P. Carvalho [2,3,*]

[1] Departamento de Engenharia Química, Instituto Superior de Engenharia de Lisboa, IPL, R. Conselheiro Emídio Navarro, 1, 1959-007 Lisboa, Portugal
[2] Centro de Química Estrutural, Faculdade de Ciências, Institute of Molecular Sciences, Universidade de Lisboa, Campo Grande, 1749-016 Lisboa, Portugal
[3] Departamento de Química e Bioquímica, Faculdade de Ciências Universidade de Lisboa, Ed.C8, Campo Grande, 1749-016 Lisboa, Portugal
* Correspondence: amartins@deq.isel.ipl.pt (A.M.); apcarvalho@fc.ul.pt (A.P.C.)

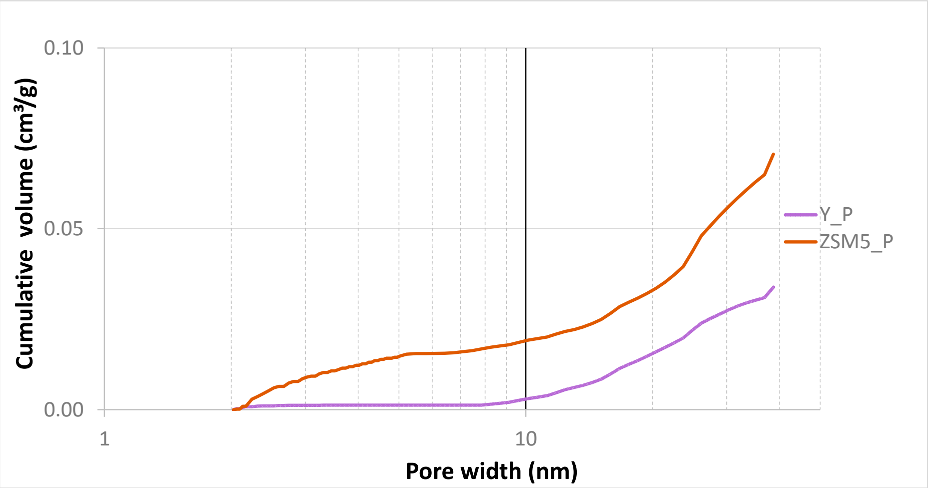

Figure S1. Mesopore size distribution for pre-treated samples Y_P and ZSM5_P.
